# Ultrasound‐Driven Piezoelectrocatalytic Immunoactivation of Deep Tumor

**DOI:** 10.1002/advs.202303016

**Published:** 2023-08-16

**Authors:** Anbang Wu, Lingdong Jiang, Chao Xia, Qingqing Xu, Bin Zhou, Zhaokui Jin, Qianjun He, Jinxiao Guo

**Affiliations:** ^1^ Department of Orthopaedics Shanghai Jiao Tong University Affiliated Sixth People's Hospital Shanghai Jiao Tong University Shanghai 200233 China; ^2^ School of Biomedical Engineering Shenzhen University Medical School Shenzhen Guangdong 518060 China; ^3^ College of Pharmacy Shenzhen Technology University Shenzhen 518118 China; ^4^ Shanghai Key Laboratory of Hydrogen Science & Center of Hydrogen Science School of Materials Science and Engineering Shanghai Jiao Tong University Shanghai 200240 China

**Keywords:** hydrogen therapy, immune activation, nanomedicine, piezoelectric catalysis, tumor immunotherapy

## Abstract

Tumor heterogeneity makes routine drugs difficult to penetrate solid tumors, limiting their therapy efficacies. Based on high tissue penetrability of hydrogen molecules (H_2_) and ultrasound (US) and the immunomodulation effects of H_2_ and lactic acid (LA), this work proposes a novel strategy of US‐driven piezoelectrocatalytic tumor immunoactivation for high‐efficacy therapy of deep tumors by piezoelectrocatalytic hydrogen generation and LA deprivation. A kind of US‐responsive piezoelectric SnS nanosheets (SSN) is developed to realize US‐triggered local hydrogen production and simultaneous LA deprivation in deep tumors. The proof‐of‐concept experiments which are executed on an orthotopic liver cancer model have verified that intratumoral SSN‐medicated piezoelectrocatalytically generated H_2_ liberates effector CD8^+^ T cells from the immunosuppression of tumor cells through down‐regulating PD‐L1 over‐expression, and simultaneous LA deprivation activates CD8^+^ T cells by inhibiting regulatory T cells, efficiently co‐activating tumor immunity and achieving a high outcome of liver tumor therapy with complete tumor eradication and 100% mice survival. The proposed strategy of US‐driven piezoelectrocatalytic tumor immunoactivation opens a safe and efficient pathway for deep tumor therapy.

## Graphical abstract

A novel concept of ultrasound‐driven piezoelectrocatalytic immunoactivation of deep tumor is proposed, and develop a piezoelectric SnS nanosheet to realize highly efficient piezoelectrocatalytic hydrogen generation and lactic acid deprivation, achieving a high outcome of tumor immunoactivation by liberating and activating effector CD8^+^ T cells as well as high‐efficacy therapy of orthotopic liver cancer.

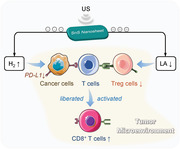

## 1 Introduction

The immunosuppression in tumor is closely related to the tumor microenvironment (TME), involving the over‐expression of many specific antibodies and chemokines.^[^ ^1^, ^2^ ^]^ Some methods of immune activation have been developed for immunotherapy, including programmed death‐1/programmed death‐ligand 1 (PD‐1/PD‐L1) immune‐checkpoint inhibition, and chemoradiotherapeutic and hyperthermal immunoactivations.^[^ ^3^, ^4^, ^5^, ^6^, ^7^ ^]^ However, the toxic side effects caused by off‐target are often unavoidable, and thus to realize the tumor‐targeted immunotherapy is of great significance for high‐efficacy and low‐toxicity tumor treatment. Among various signaling molecules, lactic acid (LA) over‐expressed in tumor plays an important role in creating an immunosuppressive microenvironment.^[^ ^8^ ^]^ Therefore, we here proposed a strategy of in situ catalytic activation of tumor immunity via catalytically oxidizing/consuming LA which was employed as a sacrificial agent in the TME.

Hydrogen inhalation has proven to be able to improve the systemic immunity of tumor patients to reduce the toxic side effects of radiotherapeutic and chemotherapeutic drugs,^[^ ^9^, ^10^ ^]^ and the local sustained supply of H_2_ can activate the immune response at the wound site to promote wound repair,^[^ ^11^ ^]^ showing an immunomodulatory potential of H_2_. In this work, we unexpectedly uncovered that H_2_ can regulate down the expression of PD‐L1 on some tumor cells such as Hepa 1–6 liver tumor cells, so we here speculated that H_2_ can achieve tumor immunotherapy by locally activating tumor immunity. In situ photocatalytic generation of H_2_ at the tumor site can realize sustainable tumor treatment,^[^ ^12^, ^13^, ^14^, ^15^, ^16^, ^17^, ^18^, ^19^, ^20^, ^21^ ^]^ but the tissue penetrability of light is quite limited, resulting in the incompetence for treating deep tumors. Therefore, we here proposed a strategy for ultrasound (US)‐driven piezoelectrocatalytic hydrogen production to activate the immunity of deep tumors. Moreover, H_2_ possesses a particularly strong ability of tissue penetration due to its smallest molecular size and low polarity,^[^ ^11^, ^22^, ^23^ ^]^ and therefore can easily penetrate into tumor for immunoactivation.

**Figure 1:**
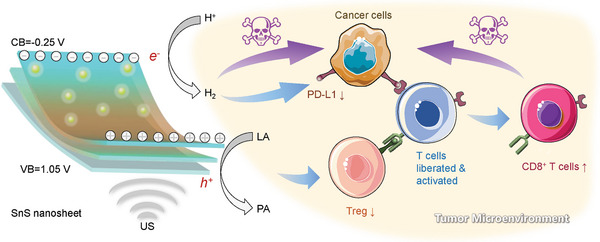
Schematic illustration of the mechanism of SnS nanosheets‐mediated piezoelectrocatalytic hydrogen generation and lactic acid deprivation for tumor immunoactivation. Several main advances are achieved. 1) Advanced concept of US‐driven piezoelectrocatalytic immunoactivation of deep tumor is proposed. 2) Advanced SSN is developed to realize the US‐driven piezoelectrocatalytic immunotherapy of deep tumor. 3) Advanced mechanisms for hydrogen generation/LA deprivation immunoactivating deep tumor are uncovered.

In this work, a kind of 2D SnS nanosheets (SSN) with excellent piezoelectric effect was developed to realize the US‐driven piezoelectric catalysis for local hydrogen production and LA deprivation at a low power density of US. On an orthotopic liver cancer model, piezoelectrocatalytically generated H_2_ and simultaneous LA deprivation co‐activated tumor immunity by liberating effector CD8^+^ T (*T*_CD8_^+^) cells from the immunosuppression of tumor cells and by inhibiting regulatory T (*T*_reg_) cells, respectively (**Figure** **1**), achieving highly efficient immunotherapy of liver cancer.

## 2 Results and Discussion

### 2.1 Synthesis and Characterization of SSN

**Figure 2:**
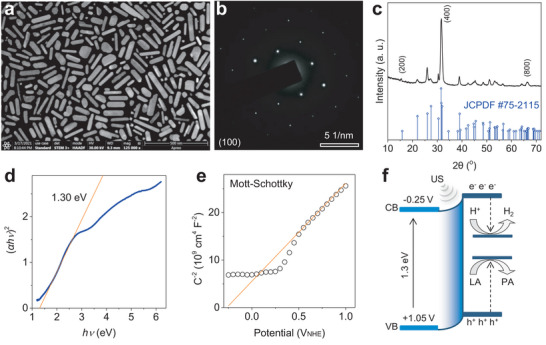
Morphology, composition, and band structure characterization of SSN. a) The STEM image, b) SEAD image, c) XRD pattern, d) curve of (*αhν*)^2^ versus *hv*, e) Mott–Schottky curve of SSN, and f) the schematic illustration of US‐driven piezoelectrocatalytic hydrogen generation and LA consumption based on SSN. NHE, normal hydrogen electrode.

In order to obtain a high piezoelectric performance, a kind of sheet‐like SnS nanoparticles (SSN) was synthesized by a solvothermal method using polyvinylpyrrolidone (PVP) as a dispersant. As observed from scanning transmission electron microscopy (STEM), the as‐prepared SSN exhibited a thin nanosheet‐like morphology (**Figure** **2a**; Figure S1a, Supporting Information). The atomic force microscopy (AFM) data further indicated that the thickness of SSN was only ≈0.8 nm (Figure S2, Supporting Information). Selected area electron diffraction (SAED), and high‐resolution TEM and X‐ray diffraction (XRD) patterns accordingly suggested that SSN was a kind of single‐crystal nanosheets with (100) orientation (Figure 2b,c; Figure S1b, Supporting Information). Dynamic light scattering (DLS) measurement showed that SSN had a hydrated diameter of ≈160 nm in support of their accumulation in liver (Figure S3, Supporting Information), and had a high stability of dispersion in the PBS solution within 7 days (Figure S4, Supporting Information). From the photocurrent–voltage curve, the bandgap of SSN was determined to be 1.30 eV (Figure 2d) in accordance with UV absorption result (Figure S5, Supporting Information). The flat band potential of SSN can be calculated by the Mott–Schottky test, from which the conduction band of SSN was determined to be −0.25 V (Figure 2e). In addition, the band structure of piezoelectric catalysts can be tilted upward by mechanical force including the US cavitation‐induced pressure in favor of hydrogen evolution. According to these results, the energy band structure of SSN was illustrated in Figure 2f. It can be found that SSN had enough high catalytic redox potentials for H^+^/H_2_ evolution and LA oxidation (LA/PA oxidation potential, 0.19 eV)^[^ ^24^ ^]^ (Figure 2f).

### 2.2 Piezoelectric Behavior and Piezoelectrocatalytic Performance of SSN

**Figure 3:**
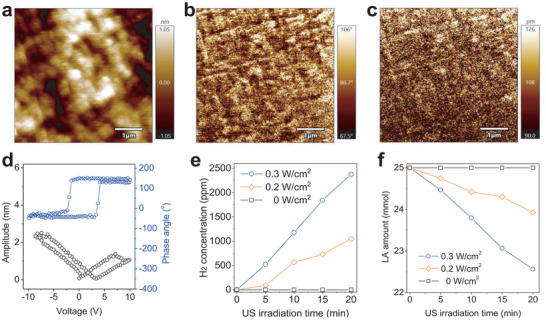
Piezoelectric property and piezoelectrocatalytic performance of SSN. a) The AFM pattern, b) corresponding PFM phase, and c) amplitude patterns, d) the piezo‐responsive phase/amplitude curves of SSN, e) US‐driven piezoelectrocatalytic hydrogen generation, and f) LA consumption at different US power densities in the aqueous solution of SSN.

Furthermore, the piezoelectric behavior of SSN was measured using piezoresponse force microscope (PFM). From the piezoelectric amplitude and phase diagrams of SSN in **Figure** **3**a‒d, a typical butterfly‐like amplitude curve was visible, and there was an ≈180° phase switch in the local circuit of piezoelectric hysteresis in the phase diagram, exhibiting a typical piezoelectric effect of SSN. Based on the confirmed‐above piezoelectric effect and proper band structure of SSN, the US‐driven piezoelectrocatalytic H_2_ generation and LA deprivation were checked in vitro at first. As shown in Figure 3e,f, SSN can indeed be excitated by a low intensity of US for H_2_ generation and LA oxidation/deprivation in a US power‐dependent way, but no hydrogen was produced and no LA was consumed in the absence of US irradiation, indicating the piezoelectrocatalytic behavior of SSN. In addition, piezoelectrocatalytic hydrogen generation was highly responsive to the irradiation of US (Figure S6, Supporting Information), reflecting the high controllability of US‐triggered hydrogen generation based on SSN in favor of on‐demand therapy.

### 2.3 In Vitro Piezoelectrocatalytic Anti‐Cancer Performance of SSN

**Figure 4:**
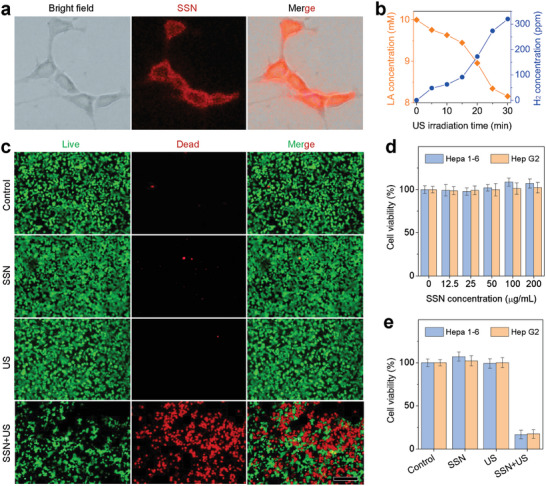
In vitro piezoelectrocatalytic and anti‐cancer performances of SSN. a) The cellular uptake behavior of SSN observed by high‐resolution Raman microscopy, b) the intracellular SSN‐mediated piezoelectrocatalytic hydrogen production and LA consumption, c) fluorescence images (scale bar, 200 µm) of Hepa 1–6 cells stained by Calcein AM/PI after different treatments, d) Hepa 1–6 and Hep‐G2 cell viabilities (*n* = 5 biologically independent samples) after incubation with various concentrations of SSN without US irradiation, and e) in vitro piezoelectrocatalytic (0.3 W cm^−2^, 10 min) anti‐cancer behaviors of SSN (200 µg mL^−1^) (*n* = 5 biologically independent samples).

The piezoelectrocatalytic behavior of SSN was further confirmed in the cell level, and the anti‐cancer performance of US‐driven piezoelectrocatalytic hydrogen generation was investigated on a liver cancer cell model (Hepa 1–6 cells). At first, the cellular uptake behavior of SSN in vitro was investigated. After SSN were co‐incubated with Hepa 1–6 cells for 4 h, and the endocytosis of SSN was imaged on a high‐resolution Raman microscope. Notably, this imaging method can truly reflect the biological behavior of SSN because of no need for fluorescence labeling on nanomaterials.^[^ ^25^ ^]^ According to the characteristic Raman peaks of SSN at 100‒250 cm^−1^ (Figure S7, Supporting Information), the Raman signal of SSN was well detected within Hepa 1–6 cells (**Figure** **4**a), indicating that SSN was internalized into the cells. Moreover, fluorescence imaging results about the ICG‐labelled SSN suggested that SSN were internalized into the cells by a lysosome pathway (Figure S8, Supporting Information). Then the intracellular SSN‐mediated piezoelectrocatalytic hydrogen production and LA deprivation were further investigated. From Figure 4b, under the stimulation of US, SSN was able to piezoelectrocatalytically produce hydrogen and consume LA simultaneously in cells, which was consistent with the experimental results in the solution (Figure 3e,f).

Since H_2_ has an anti‐cancer effect,^[^ ^26^ ^]^ the in vitro piezoelectrocatalytic anti‐cancer performances of SSN on hepatocellular carcinoma cells were investigated. In order to evaluate the cell‐killing effect visually, Calcein AM/PI was used to stain living and dead cells with green and red after treatment, respectively. As shown in Figure 4c, neither only SSN treatment nor only US irradiation cause obvious Hepa 1–6 cell death, while the SSN+US treatment (0.3 W cm^−2^, 10 min) significantly induced their death in a US intensity‐ and time‐dependent way (Figure S9, Supporting Information) in accordance with CCK‐8 results (Figure 4d,e), reflecting the anti‐cancer effect of piezoelectrocatalytically generated H_2_. Moreover, the anti‐cancer outcomes of piezoelectrocatalytic therapy on Hepa 1–6 cells and Hep‐G2 cells were similar (Figure 4e). In addition, from Figure 4d and Figure S10 (Supporting Information), SSN (12.5‒200 µg mL^−1^) did not show obvious cytotoxicity to Hepa 1–6 cells, Hep‐G2 cells, and LO2 cells in the absence of US irradiation, suggesting high cytocompatibility of SSN.

The diffusion of therapeutic agents in tumor is an important factor affecting their anti‐cancer efficacy. In order to evaluate the diffusion ability of hydrogen molecules, a hydrogen probe (NDI‐N_3_/Pd@MSN‐PEG) was employed to detect the hydrogen production and diffusion in tumor spheroids.^[^ ^22^ ^]^ The hydrogen probe was first incorporated into the Hepa 1–6 tumor spheroids during the formation of spheroids, and then SSN were co‐incubated with Hepa 1–6 tumor spheroids for 6 h followed by US irradiation (0.3 W cm^−2^, 10 min) and then fluorescence imaging on confocal microscope. The results in Figure S11 (Supporting Information) showed that green/blue fluorescence quickly appeared on the whole tumor spheroid, and the fluorescence ratio (G/B) gradually increased within 14 min under US irradiation, indicating that piezoelectrocatalytically generated hydrogen can rapidly diffuse into the interior of the tumor spheroid, which would greatly favor for intratumoral immunomodulation.

### 2.4 In Vitro Piezoelectrocatalytic Immunomodulation Behavior

**Figure 5:**
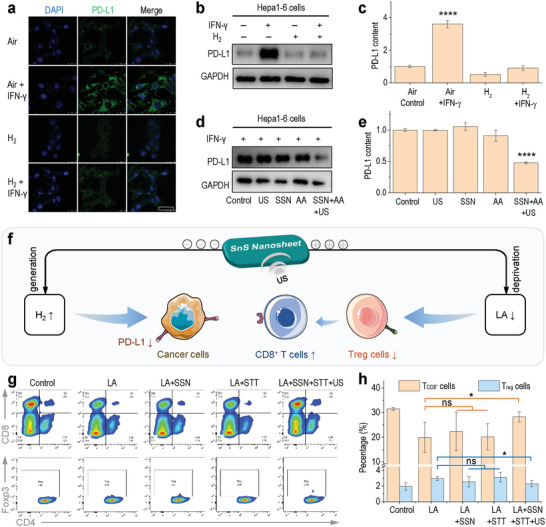
In vitro immunomodulation effects of US‐driven piezoelectrocatalytic hydrogen generation and LA deprivation. a) Confocal fluorescence images of Hepa 1–6 cells after various treatments (Scale bar, 50 µm), where Air and H_2_ groups represented incubation in general and hydrogen incubators, respectively, and b) the corresponding WB patterns and c) quantitative analysis for distinguishing the effect of H_2_ on PD‐L1 expression, d) the WB patterns and e) corresponding quantitative analysis of PD‐L1 expression on Hepa 1–6 cells treated with H_2_ generated by SSN‐mediated US‐driven piezoelectrocatalysis, the schematic illustration showing the effects of piezoelectrocatalytic hydrogen generation and f) LA deprivation on cancer and T cells, g) flow cytometry of T cells incubated without (control) or with LA, and h) the comparison of the proportion of *T*_CD8_^+^ and *T*_reg_ cells among various treatments. *P* values were calculated by a two‐tailed Student's *t*‐test method (^*^ *p* < 0.05; ^****^ *p* < 0.0001; ns, no significant difference).

Based on the role of LA in the creation of tumor immunosuppressive microenvironment, the in vitro immunological effects of US‐driven piezoelectrocatalytic hydrogen generation and LA deprivation were investigated, respectively. At first, Hepa 1–6 cells were incubated in a specific hydrogen incubator and in a general incubator, respectively, to explore the effect of hydrogen on the immunophenotype of cancer cells. Unexpectedly, from immunofluorescence and Western blotting assays (**Figure** **5a‒c**), we observed the distinct inhibition of H_2_ to PD‐L1 over‐expression on IFN‐γ‐irritated Hepa 1–6 cells in a simulated tumor microenvironment for the first time. It meant that H_2_ is possibly able to activate the immunologically inhibited T cells in tumor for immunotherapy. We speculated that the PD‐L1 expression‐inhibiting effect of H_2_ might be derived from the respiration‐inhibiting and energy‐regulating functions of H_2_ against cancer cells. In order to exclude the effect of LA deprivation, ascorbic acid (AA) was used as a hole‐sacrificial agent because of its high cytocompatibility and high reducibility. It can be found from Figure 5e that the SSN‐mediated piezoelectrocatalytically generated H_2_ (200 µg mL^−1^ SSN + 10 µm AA + 0.3 W cm^−2^ 10 min US) can also effectively inhibit the over‐expression of PD‐L1 on IFN‐γ‐irritated Hepa 1–6 cells. Therefore, the US‐driven piezoelectrocatalytic hydrogen generation based on SSN held a potential for tumor immunoactivation by intercepting the PD‐L1/PD‐1 pathway (Figure 5f).

On the other hand, Na_2_S_4_O_6_ (STT) was used as an electron‐sacrificial agent to investigate the immunological effect of individual LA deprivation by SSN‐mediated piezoelectric catalysis because of its high cytocompatibility and high oxidability. LA, an overexpressed substance in the TME, is also responsible for immunosuppression by inhibiting the activity of *T*_reg_ cells (Figure 5c). T lymphocytes, including both the tumor‐killing CD8^+^ T (*T*_CD8_^+^) cells and the immunosuppressive *T*_reg_ cells, were extracted from the spleen of C57 mice using a CD3 sorting kit, and then cultured with LA (10 mm) for 24 h followed by piezoelectrocatalytic LA deprivation (200 µg mL^−1^ SSN + 100 µm STT + 0.3 W cm^−2^ 10 min US). From Figure 5g,h, LA induced the increase of *T*_reg_ cells and the decrease of *T*_CD8_^+^ cells, exhibiting a typical immunosuppression effect. By comparison, the piezoelectrocatalytic LA deprivation inhibited the LA‐induced *T*_reg_ cells and also activated the LA‐suppressed *T*_CD8_^+^ cells, playing a function of immunoactivation. Therefore, the piezoelectrocatalytic LA deprivation based on SSN held a potential for tumor immunoactivation by the *T*_CD8_^+^/*T*_reg_ pathway (Figure 5f).

### 2.5 In Vivo Piezoelectrocatalytic Immunoactivation and Therapy Outcomes

Furthermore, the immunoactivation and therapy outcomes of SSN‐mediated US‐driven piezoelectrocatalysis were checked in vivo on a deep tumor model of orthotopic hepatoma. The orthotopic hepatoma model was built by implanting liver cancer cells (10^7^ Hepa 1–6‐Luc cells) into the liver of C57BL/6 mice.^[^ ^27^ ^]^ After 10 days, fluorescence imaging post intraperitoneal injection of 150 mg kg^−1^ D‐luciferin was used to determine the success of model. Then, SSN (100 µL, 10 mg kg^−1^) was intravenously injected into the Hepa 1–6‐Luc tumor‐bearing mice followed by tumor‐directing US irradiation (3 W cm^−2^, 10% duty cycle, 10 min) once a day for one week. Before that, high tissue penetration of the used US was confirmed to be competent for piezoelectrocatalytic therapy of liver cancer (Figure S12, Supporting Information). After one month, the liver was extracted for immunohistochemical analysis.

**Figure 6:**
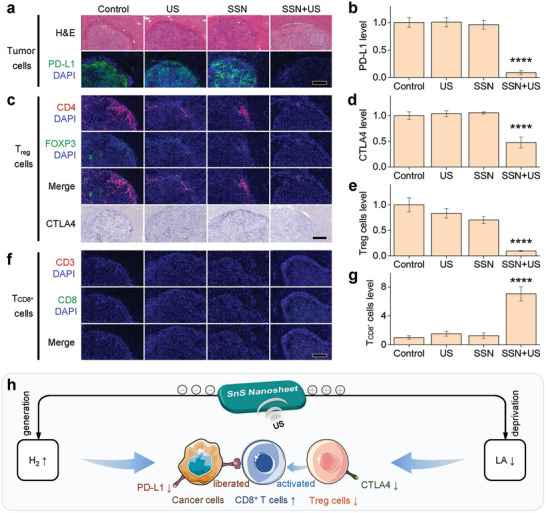
In vivo US‐driven piezoelectrocatalytic immunoactivation of liver tumor. a) Immunohistochemical analysis of PD‐L1 in tumor cells, c) *T*_reg_ (CD4, FoxP3, and CTLA4) cells, and f) *T*_CD8_^+^ (CD3, CD8) cells in extracted tumor tissue at the end of treatment, b,d,e,g) corresponding quantitative results, and h) the schematic illustration of tumor immunoactivation of piezoelectrocatalytic hydrogen generation and LA deprivation. *p‐*values were calculated by the two‐tailed Student's *t*‐test method (^****^ *p* < 0.0001; ns, no significant difference). All the scale bars in insets (a,c), and (f) correspond to 500 µm.

Firstly, H&E sections showed that the nuclei of tumor cells in the SSN+US group were obviously reduced compared to other three groups, indicating an efficient suppression of US‐driven piezoelectrocatalytic therapy to tumor growth. Notably, US‐driven piezoelectrocatalytic therapy with SSN+US caused a significant downregulation of PD‐L1 in tumor (**Figure** **6**a,b), owing to the contribution of piezoelectrocatalytically generated hydrogen (Figure 5a‒e). The co‐localization of CD4 red fluorescence and Foxp3 green fluorescence showed that US‐driven piezoelectrocatalytic therapy with SSN+US led to significant decreases of *T*_reg_ cell number (Figure 6c,d) and CTLA4 level (Figure 6c,e) and a clear increase of *T*_CD8_^+^ cell number (Figure 6f,g), which should be contributed to piezoelectrocatalytic LA deprivation in tumor as indicated in vitro (Figure 5g,h). Therefore, SSN‐mediated US‐driven piezoelectrocatalytic therapy activated Hepa 1–6 tumor immunity from two aspects by hydrogen generation and LA deprivation, as illustrated in Figure 6h. On the one hand, piezoelectrocatalytically generated hydrogen inhibited the PD‐L1 expression on tumor cells, which can liberate the immunosuppressed *T*_CD8_^+^ cells. On the other hand, piezoelectrocatalytic LA deprivation suppressed *T*_reg_ cells to activate *T*_CD8_^+^ cells. The combined immunoactivation would support piezoelectrocatalytic immunotherapy of hepatoma.

**Figure 7:**
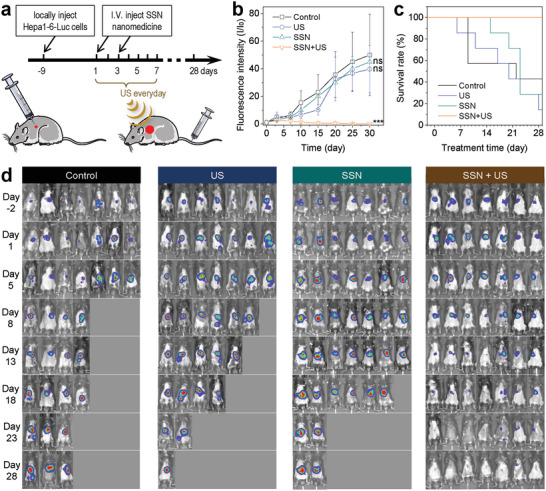
In vivo tumor therapy efficacy of SSN‐mediated US‐driven piezoelectrocatalysis. a) The orthotropic liver cancer model establishment and treatment procedure, b) the tumor fluorescence change during treatment, c) the survival rate of liver tumor‐bearing mice after treatment, and d) fluorescence photographs of liver tumor‐bearing mice during treatment (*n* = 7 biologically independent samples). *P* values were calculated by the two‐tailed Student's *t*‐test method ^(***^ *p* < 0.001; ns, no significant difference).

Before treatment, the ICP measurement of Sn was carried out to evaluate the biodistribution of SSN in the Hepa 1–6 tumor‐bearing mice. As shown in Figure S13 (Supporting Information), the SSN nanocatalysts can effectively accumulate in the tumor‐bearing liver with a high accumulation efficiency (≈65%) after intravenous injection in a passive targeting way. Due to the spatial limitation of deep tumors, Hepa 1–6‐Luc cells (firefly luciferase‐expressing Hepa 1–6 liver cancer cells) were chosen to construct the tumor model for facile real‐time monitoring of tumor growth in vivo. The substrate D‐luciferin was injected intraperitoneally into the tumor‐bearing mice and then generated bioluminescence when entering Hepa 1–6‐Luc tumor cells, which allowed us to indirectly determine the tumor size through animal fluorescence imaging. The Hepa 1–6‐Luc tumor‐bearing mice were divided into four groups (*n* = 7): control group, SSN group, US group, and SSN+US group. The PBS solution of SSN (2 mg mL^−1^, 100 µL) was intravenously injected into the tumor‐bearing mice in the SSN+US group on days 1 and 3 followed by US irradiation (3 W cm^−2^, 10% duty cycle, 10 min) once a day for one week (**Figure** **7**a), and the tumor bioluminescence, body weight, and survival rate were recorded for 28 days.

The bioluminescence imaging in Figure 7b,d showed that in the control group, liver tumor quickly grew up, leading to a sharp decline in the survival rate of the tumor‐bearing mice (42.8% on day 28). Individual US treatment did not bring significant benefit to tumor inhibition and mice survival rate, but US‐driven piezoelectrocatalytic therapy with SSN+US remarkably inhibited the growth of tumors, and the survival rate of treated mice maintained at 100% during the whole treatment of 28 days (Figure 7b‒d). Especially, from bioluminescence imaging of the liver extracted at the end of treatment in Figure S14 (Supporting Information), US‐driven piezoelectrocatalytic therapy with SSN+US almost completely eradicated the orthotopic liver tumors. Furthermore, immunohistochemical analysis of tumor tissue sections with Ki67 and Tunel stainings indicated that US‐driven piezoelectrocatalytic therapy with SSN+US induced the extensive apoptosis of tumor cells (Figure S15, Supporting Information) and significantly inhibited the proliferation of tumor cells (Figure S15, Supporting Information), but other two groups did not. These excellent outcomes of US‐driven piezoelectrocatalytic therapy were possibly attributed to both combined tumor immunoactivation effects of hydrogen generation and LA deprivation and the tumor‐killing effect of hydrogen molecules as mentioned above in vitro and in vivo.

In addition, neither obvious decrease of body weight nor histopathological change of major organs in all the groups was observed, suggesting high biosafety of the US power adopted and SSN (Figures S17 and S18, Supporting Information). Moreover, from Figures S19 and S20 (Supporting Information), compared with the control group, there was no significant difference of hematological parameters among all the experimental groups, and the biochemical analysis also showed that SSN had no significant adverse effect on the liver and renal functions of mice. These results indicated that both SSN and US had high biocompatibility, ensuring the biosafety of US‐driven piezoelectrocatalytic therapy with SSN+US.

## 3 Conclusion

In summary, we developed a 2D SSN nanocatalyst to realize US‐driven piezoelectrocatalytic immunoactivation of deep tumor. Under ultrasonic stimulation, the SSN nanocatalyst with piezoelectric effect exhibited controlled catalytic hydrogen generation and LA oxidation/deprivation to inhibit the liver tumor growth by immune regulation of TME. In vitro and in vivo experiments demonstrated that hydrogen molecules were able to effectively down‐regulate the expression of PD‐L1 on Hepa 1–6 cells and the deprivation of LA in tumor reduced the activity of *T*_reg_ cells, thereby together achieving the goal of activating tumor immunity. Taking advantage of US and H_2_ in tissue penetration, the nanocatalyst ensured efficient deep tumor therapy by immunoactivation.

## 4 Experimental Section

### Synthesis of SnS Nanosheets (SSN)

SSN were synthesized by the solvothermal method. Na_2_S·9H_2_O (776 mg) was first dissolved in glycol (10 mL), followed by the addition of SnCl_2_·2H_2_O (427 mg) and PVP (50 mg). The reaction solution was poured into a polytetrafluoroethylene lined autoclave (50 mL) and then heated to 160 °C for reaction for 2 h. The solution was cooled to room temperature, and then the SSN particles were collected by centrifugation, followed by washing for three times with anhydrous ethanol and deionized water, respectively.

### Characterization of SSN

The morphology and size of SSN were characterized by SEM (APREO, FEI) and TEM (JEM‐2100F). The hydrodynamic particle size of SSN was measured with a DLS analyzer (Malvern Zetasizer Nano ZS90). The composition of SSN was determined by Fourier transform infrared spectroscopy (FTIR) on a Thermo‐Nicolet Nexus 670 ATR‐IR spectrometer. The UV absorption spectra were recorded on a Genesys 10S UV–vis spectrophotometer.

### Detection of Hydrogen Production In Vitro

The catalytic hydrogen production in solution was measured by gas chromatograph (GC). Briefly, a solution containing 2 mg mL^−1^ of SSN and 10 mm of LA in a sealed bottle was stimulated by US at different power densities and for different time durations, and then the above air was collected for measurement of hydrogen content by GC. Hydrogen content at the same time point was tested for three times with parallel samples (*n* = 3).

For the measurement of hydrogen generated in cells, Hepa 1–6 cells were co‐incubated with 200 µg mL^−1^ of SSN for 12 h. After digestion, the cells were collected by centrifugation and suspended in 1 mL PBS solution containing 10 mm LA. The mixed solution was then sealed in a little bottle for US irradiation at different power densities and for different time durations, followed by GC measurement of produced hydrogen amount.

### Measurement of Cellular Uptake of SSN

The cellular uptake of SSN was evaluated by a Raman technique based on the unique Raman signals of SSN. Briefly, Hepa 1–6 cells (1 × 10^5^) were seeded in a confocal dish, and cultured with SSN (100 µg mL^−1^) for 4 h. After that, the culture medium was replaced with PBS, and the cellular uptake behavior was observed on a Raman microscope (Thermo Scientific, DXR3xi Raman Imaging Microscope).

For the convenience to observe the cellular uptake by fluorescence imaging, SSN were labeled with a near‐infrared fluorescent dye ICG by reaction with ICG‐PEG‐SH based on the Sn–S coordination. Hepa 1–6 cells (1 × 10^5^) were seeded in a confocal dish and cultured with the ICG labeled SSN (100 µg mL^−1^) for 4 h. After that, the lysosomes and nuclei of Hepa 1–6 cells were stained with Lyso‐Tracker Green and DAPI, respectively. After the removal of excessive dyes and nanoparticles by washing, Hepa 1–6 cells were observed on a confocal laser scanning microscope (ZEISS, LSM880).

### Detection of LA Deprivation In Vitro

The simulated solution containing SSN (200 µg mL^−1^) and LA (10 mm) was irradiated by US at different power densities (0, 0.2, 0.3 W cm^−2^) for different time periods (5‒30 min). SSN in the solution were removed by centrifugation, and then the LA concentration in the supernatant was detected by a LA assay kit.

For the measurement of intracellular deprivation of LA, Hepa 1–6 cells were co‐incubated with SSN (200 µg mL^−1^) for 12 h. After digestion, the cells were collected by centrifugation and resuspended in LA solution (10 mm, 1 mL). The solution was then stimulated by US at different power densities for different time periods. The cells and nanoparticles were removed by centrifugated, and then the LA concentration in the supernatant was detected by the LA assay kit (*n* = 3).

### In Vitro Cytotoxicity Assessment

Hepa 1–6 and HepG2 cells were individually seeded into 96‐well cell culture plates at the density of 1 × 10^4^ cells/well, and incubated with various concentrations of SSN for 12 h at 37 °C in a humidified 5% CO_2_ atmosphere. Cells were irradiated with 0.3 W cm^−2^ US for 10 min from the bottom of plates. After incubation for another 24 h, the standard CCK‐8 assay was executed to determine cell viability by collecting the absorbance at 450 nm on a microplate reader (BioTek).

### Measurement of Hydrogen Penetration in MCSs

Multicellular spheroids (MCSs) were constructed to evaluate the hydrogen penetration capacity for simulation of hydrogen penetration in tumor tissue. Hepa 1–6 cells were mixed with matrigel and then seeded in a matrigel coated 96 well plate with a glass bottom at the density of 1000 cells/well, followed by addition of a hydrogen nanoprobe (NDI‐N3/Pd@MSN‐PEG). When the cells grew into tight MCSs, 100 µg mL^−1^ of SSN were co‐incubated with the MCSs for 6 h. After stimulated by US for 10 min, the MCSs were imaged by confocal laser scanning microscopy (LEICA‐SP8).

### In Vivo Tumor‐Targeted Delivery and Biodistribution Measurement

All the in vivo experiments were carried out by following the protocols approved by the Animal Care and Use Committee of Shenzhen University. The Hepa 1–6‐Luc tumor‐bearing mouse model was established by injecting 1×10^7^ Hepa 1–6‐Luc cells (dispersed in 20 µL Matrigel) into the liver of a male C57BL/6 mouse (≈20 g, purchased from Guangdong Medical Laboratory Animal Center). After implantation for 10 days, 100 µL PBS solution of SSN (10 mg kg^−1^) was intravenously injected into the Hepa 1–6‐Luc tumor‐bearing mice. Main organs (heart, liver, spleen, lung, and kidney) and tumors were extracted at fixed time points (1, 2, 4, 12, and 24 h) post‐injection. These organs were weighted and then completely digested with aqua regia, heated to dryness, and finally diluted with deionized water. The quantitative analysis of Sn was determined by ICP‐AES to measure the biodistribution of SSN in vivo (*n* = 3).

### In Vivo Tumor Therapy Assessment

For Hepa 1–6‐Luc tumor therapy, when tumor volume grew up to ≈80 mm^3^ according to tumor fluorescence imaging results after the model was built for 10 days, the treatment was performed (designated as day 1). The tumor‐bearing mice were randomly divided into four groups (*n* = 7), including PBS group, US group, SSN group, and SSN+US group. The PBS solution of SSN (10 mg kg^−1^, 100 µL) was intravenously injected on days 1 and 3 followed by US irradiation (3 W cm^−2^, 10% duty cycle, 10 min) upon tumor sites once a day for one week. The bioluminescence intensity of tumors was monitored by a IVIS spectrum, and *I*/*I*_0_ was used to reflect the change in relative tumor volume during treatment. Body weight and relative tumor volume of each mouse were recorded. The mice with tumor overgrowth were humanly executed during treatment according to animal fluorescence imaging results. After 28 days of treatment, all the mice were humanely executed. Main organs (heart, liver, spleen, lung, and kidney) and tumors were extracted for histological analysis.

### Detection of PD‐L1 Expression on Tumor Cells

To investigate the effect of hydrogen on the expression of PD‐L1 on tumor cells, Hepa 1–6 cells were seeded into 6‐well plates with glass bottom at a density of 1×10^5^ cells/well, and cultured for 24 h with or without 100 ng mL^−1^ IFN‐γ in a hydrogen incubator or normal incubator, respectively. The expression of PD‐L1 protein on Hepa 1–6 cells receiving different treatments was determined by cell immunofluorescence method and by observation under confocal microscope.

Hepa 1–6 cells were seeded into 6‐well cell culture plates, and grew up to ≈80% confluency at 37 °C in a humidified 5% CO_2_ atmosphere. After that, the cells were further incubated with IFN‐γ (100 ng mL^−1^) for 24 h, and then with SSN for another 12 h followed by irradiation with US (3 W cm^−2^, 10% duty cycle) for 10 min. After protein extraction using cell lysate, PD‐L1 protein expression was detected by Western blot.

### Assessment of Liver/Kidney Functions and Hemotoxicity

Healthy C57BL/6 mice were randomly divided into four groups (*n* = 3) followed by intravenous injection with PBS (100 µL) or PBS solution of SSN (100 mg kg^−1^, 100 µL). The mice in US and SSN+US groups were then subjected to US irradiation (3 W cm^−2^, 10% duty cycle, 10 min) toward tumors. After 2 weeks, the blood of mice was collected and assessed by using a biochemical analyzer (iMagic‐M7) and a blood cell analyzer (BC‐31s, Mindray).

### In Vivo Immunological Evaluation

To confirm the immunological effects, immune changes in vivo were investigated on the immunofluorescence sections of liver tumors. Tissue sections were co‐localized with CD3 antibody and CD8 antibody to stain CD8^+^ T cells, and co‐localized with CD4 antibody and Foxp3 antibody to stain *T*_reg_ cells. And the PD‐L1 level in tumor tissues was localized with PD‐L1 antibody.

### In Vitro Immunological Evaluation of T cells

T cells were isolated from C57BL/6 mice by a mouse CD90.2 positive selection kit, and then seeded into 6‐well cell culture plates at the density of 1×10^6^ cells/well. The cells were divided into five groups: normal culture group, high LA concentration (10 mm) group, and LA+SSN (200 µg mL^−1^) group, LA+STT group, and LA+SNN+STT+US (3 W cm^−2^, 10% duty cycle) group (*n* = 3). two groups, normal culture group and high LA concentration (10 mm) group (*n* = 3), and then incubated for 24 h at 37 °C in a humidified 5% CO_2_ atmosphere. Then, CD4 antibody and FoxP3 antibody were used to co‐locate *T*_reg_ cells, while CD8^+^ T cells were located by CD8 antibody.

### Statistical Analysis

Statistical analysis was performed with GraphPad Prism 8. All the experimental data were presented as the mean ± SD. The two‐tailed Student's *t*‐test was used for the comparison between two groups. The value of *p* < 0.05 was considered statistically significant (^*^ *p* < 0.05; ^**^ *p* < 0.01; ^***^ *p* < 0.001; ^****^ *p* < 0.0001; ns, no significant difference).

## Conflict of Interest

The authors declare no conflict of interest.

## Supporting information

Supporting Information

## Data Availability

The data that support the findings of this study are available from the corresponding author upon reasonable request.
