# Supplementary material for: Ultrasound‐Driven Piezoelectrocatalytic Immunoactivation of Deep Tumor
Source: Adv Sci (Weinh). 2023 Aug 16;10(28):2303016. doi: 10.1002/advs.202303016 (PMC10558630; doi:10.1002/advs.202303016)
Supplement: Supplementary file 1 — Supporting Information [file ADVS-10-2303016-s001.pdf]

ADVANCED SCIENCE Open Access

Supporting Information

for *Adv. Sci.*, DOI 10.1002/advs.202303016

Ultrasound-Driven Piezoelectrocatalytic Immunoactivation of Deep Tumor

Anbang Wu, Lingdong Jiang, Chao Xia, Qingqing Xu, Bin Zhou, Zhaokui Jin, Qianjun He and Jinxiao Guo**

Supporting Information

Ultrasound-driven piezoelectrocatalytic immunoactivation of deep tumor

Anbang Wu,[1,2,†] *Lingdong Jiang,*[2,3,†] *Chao Xia,*[2] *Qingqing Xu,*[2] *Bin Zhou,*[2] *Zhaokui Jin,*[2]
Qianjun He,[2,4,]* *Jinxiao Guo,*[1,]*

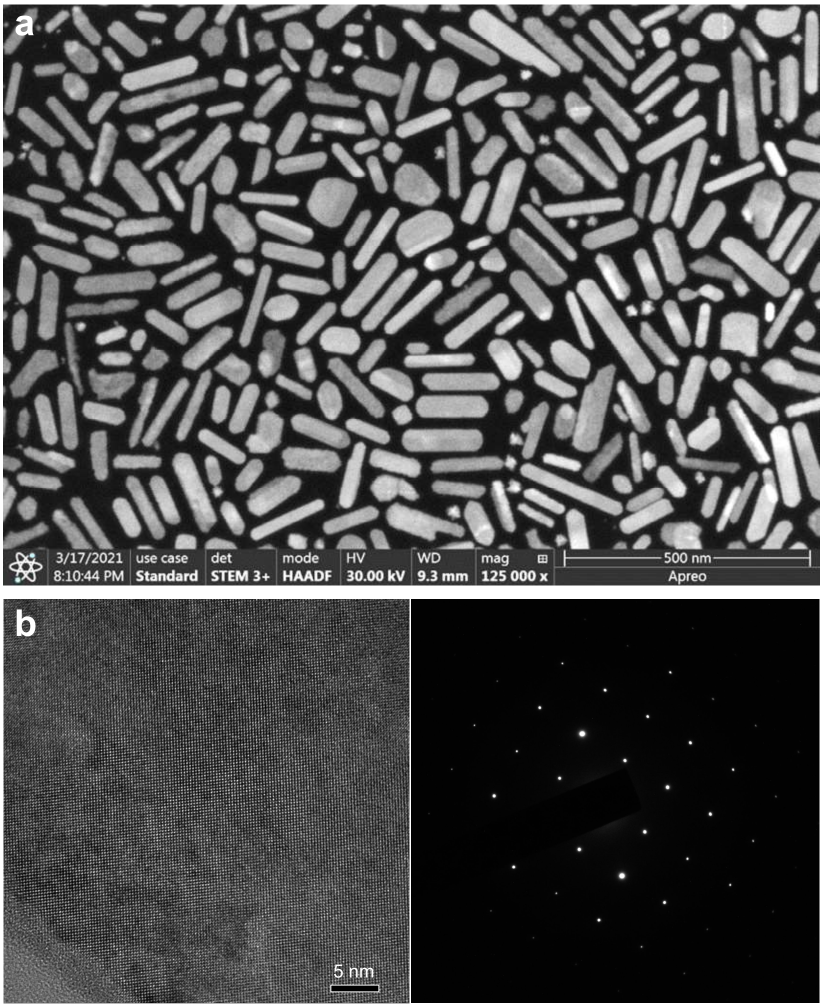

Figure S1. HADDF image (**a**), high-resolution TEM image (**b**), and SEAD pattern (**c**) of SSN.

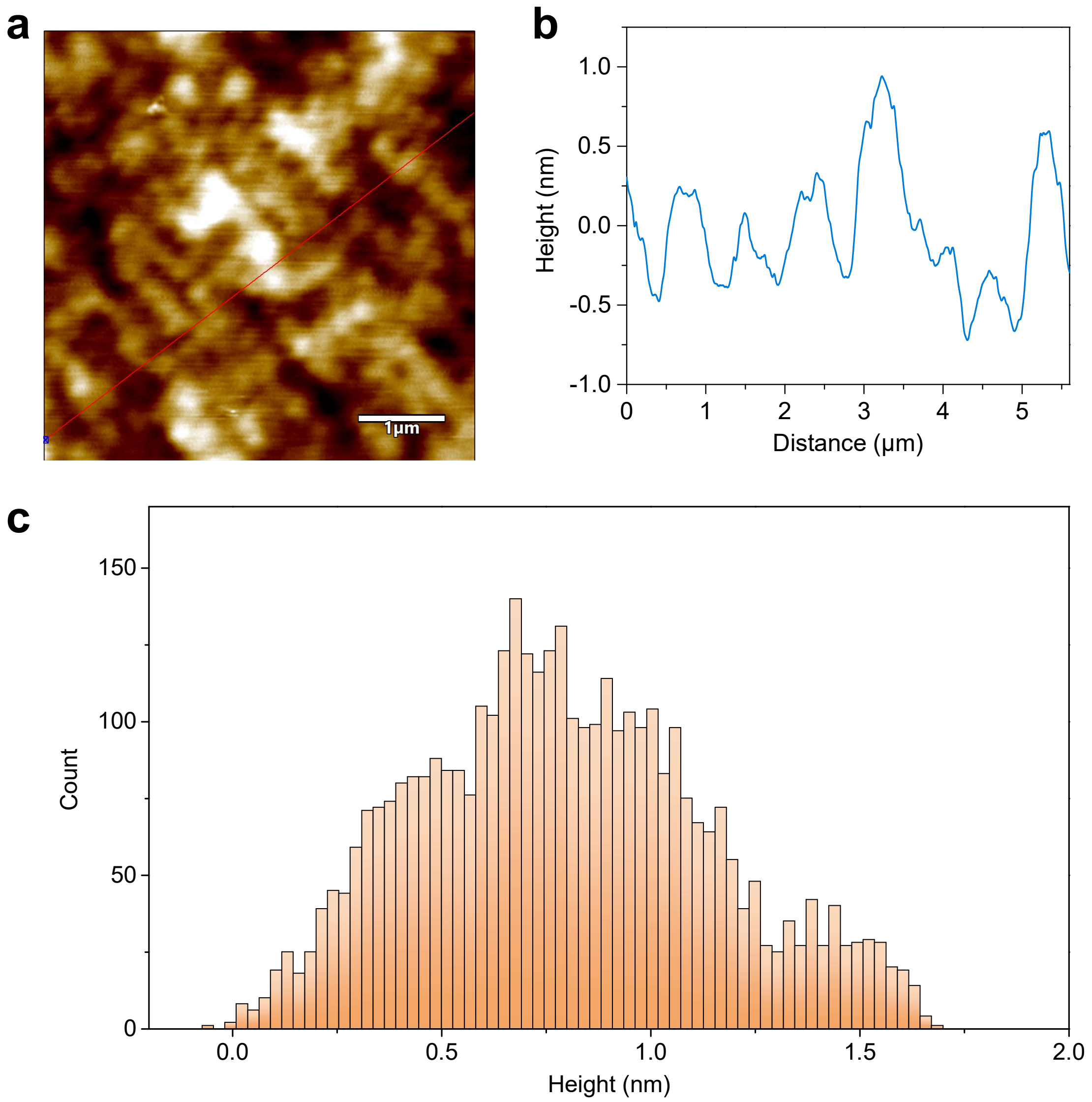

Figure S2. AFM image of SSN (**a**), corresponding height pattern (**b**) and height statistics (**c**).

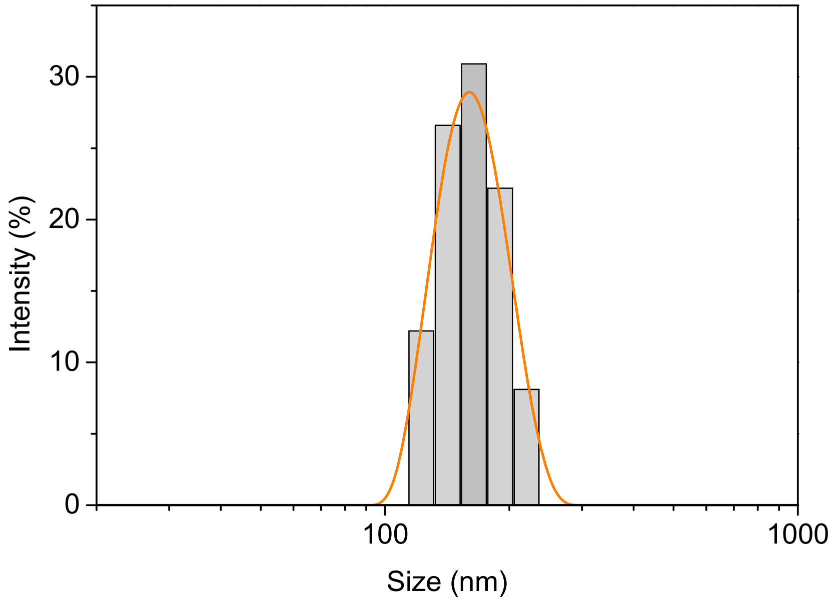

Figure S3. DLS pattern of the aqueous solution of SSN.

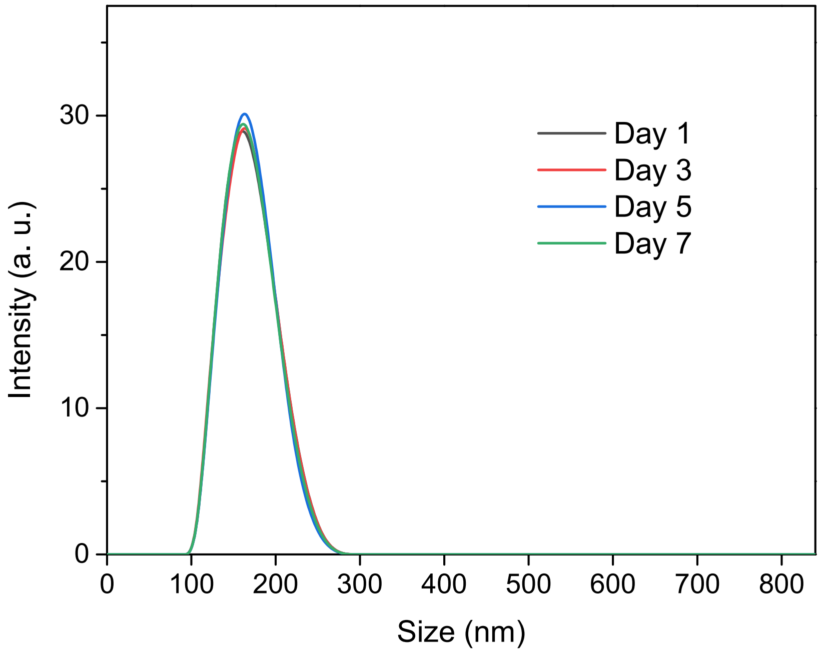

Figure S4. DLS patterns of the aqueous solutions of SSN for different dispersion time durations.

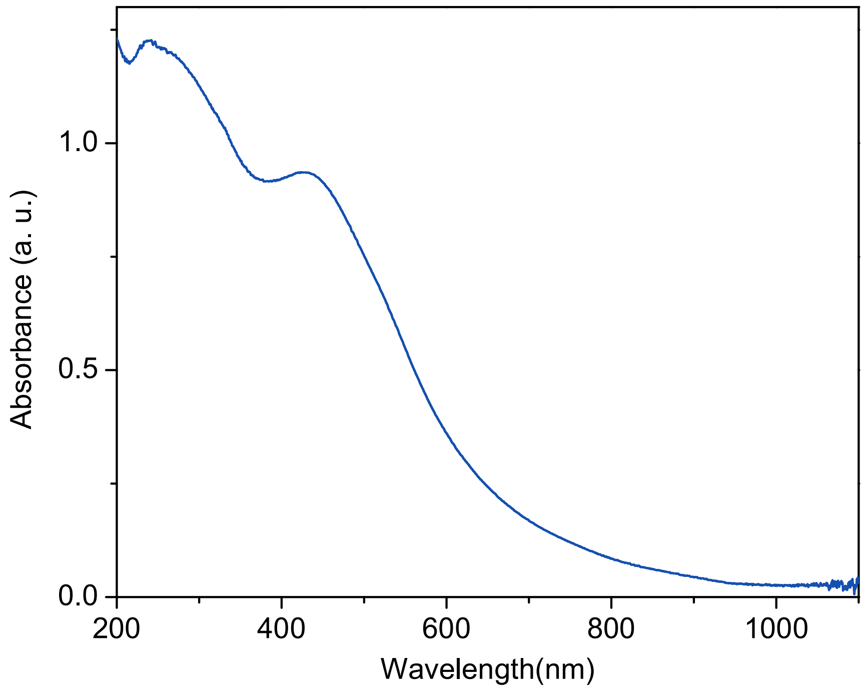

Figure S5. UV absorption spectrum of SSN.

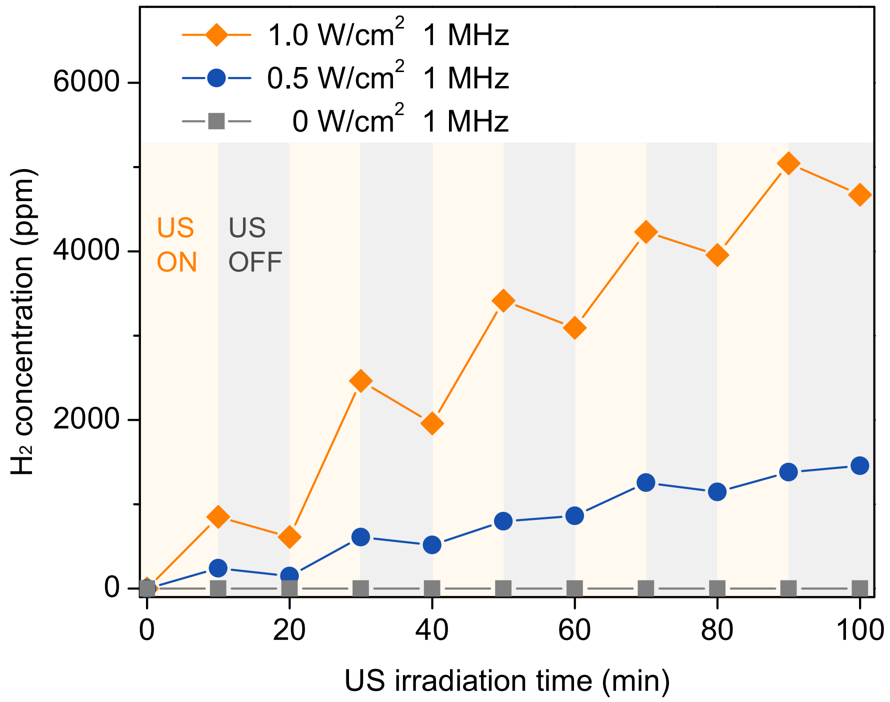

Figure S6. US controllability of SSN for catalytic hydrogen generation in the aqueous solution of LA (10 μM) under the irradiation of US at various power densities (0, 0.5 and 1.0 W/cm^2).

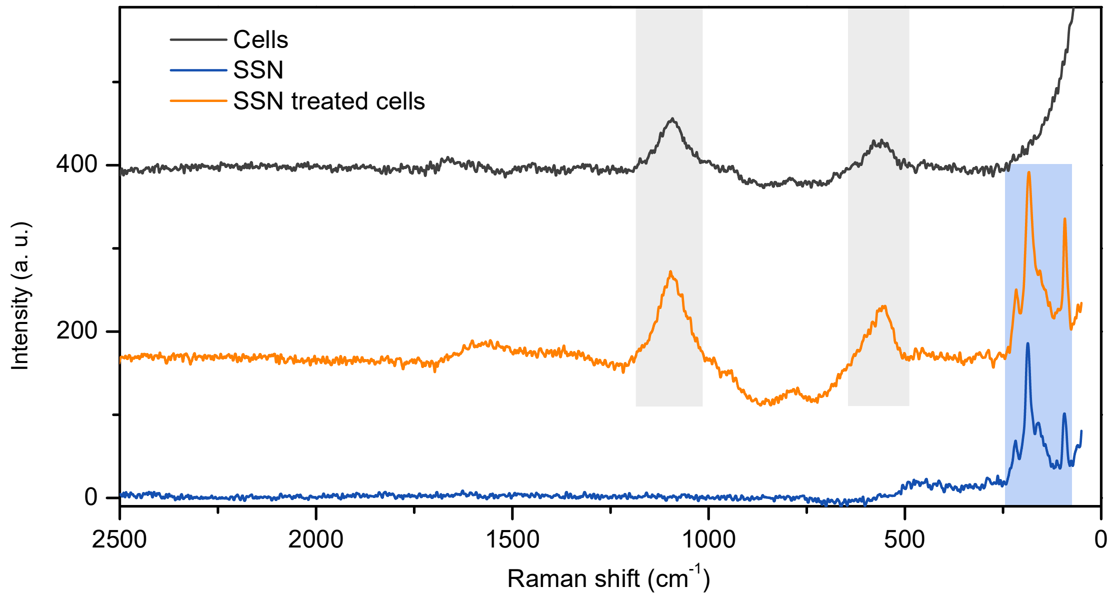

Figure S7. Raman spectra of SSN, Hepa 1-6 cells, and SSN-treated Hepa 1-6 cells.

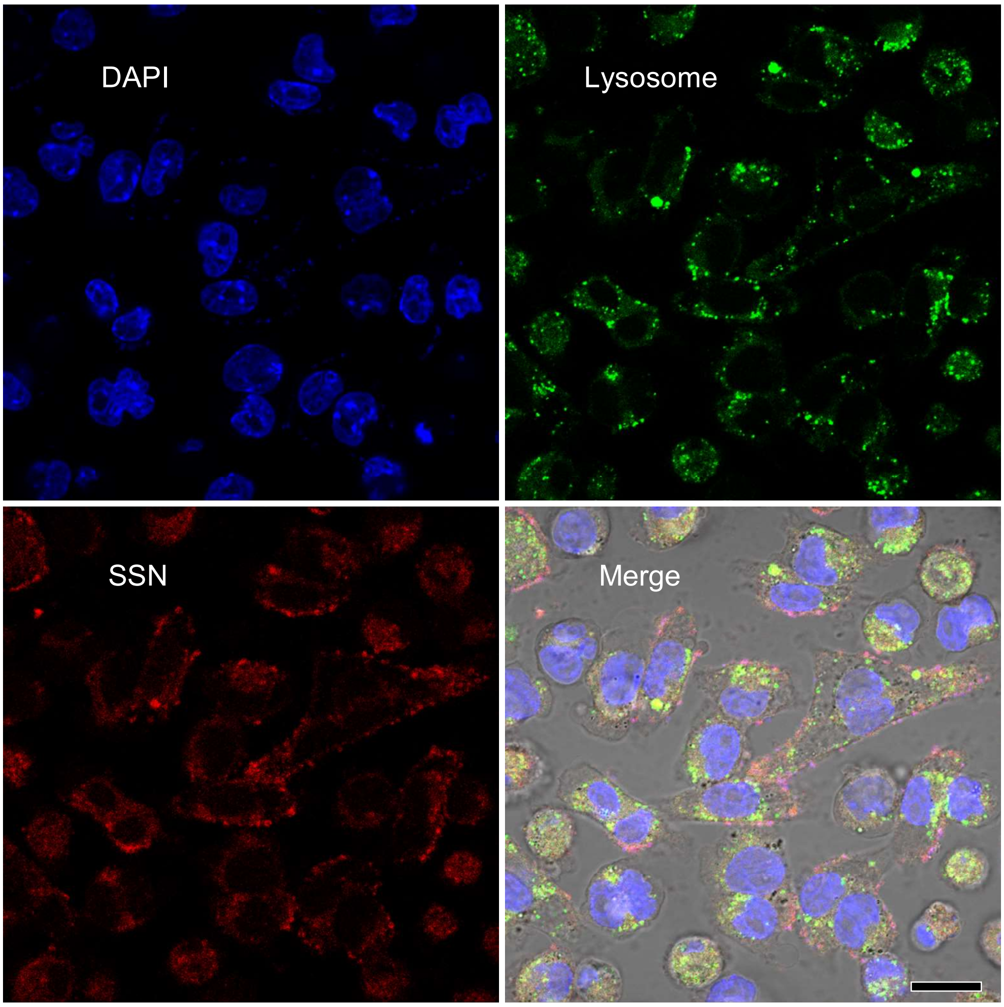

Figure S8. Confocal fluorescence images of Hepa 1-6 cells after incubation with SSN for 4 h. Scale bar, 20 μm.

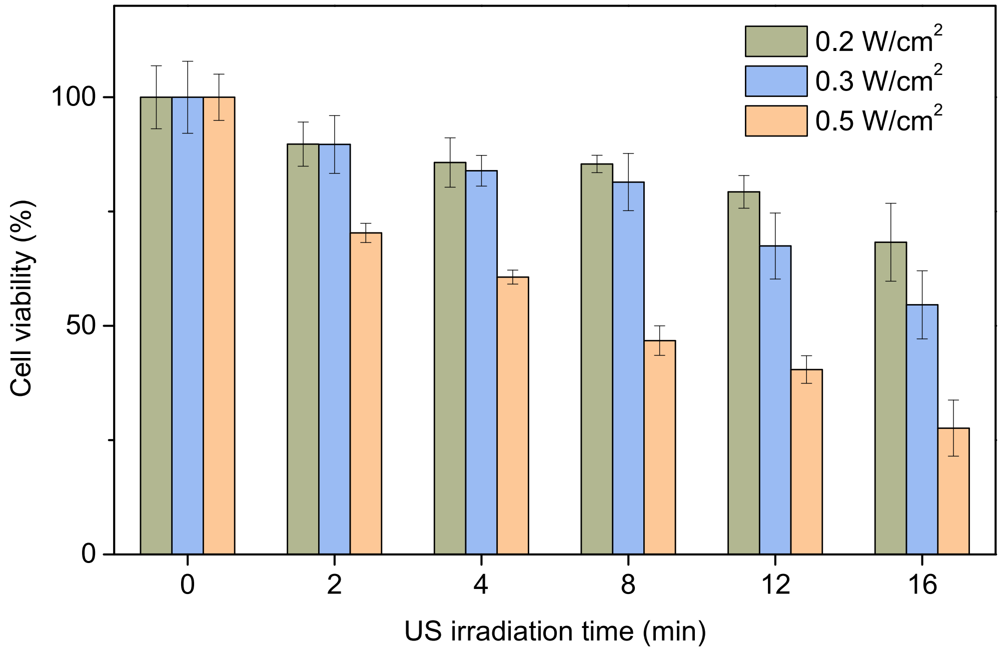

Figure S9. The cytotoxicity of SSN under irradiation of US at various power densities (0.2, 0.3 and 0.5 W/cm^2) against Hepa1-6 cells (n = 5).

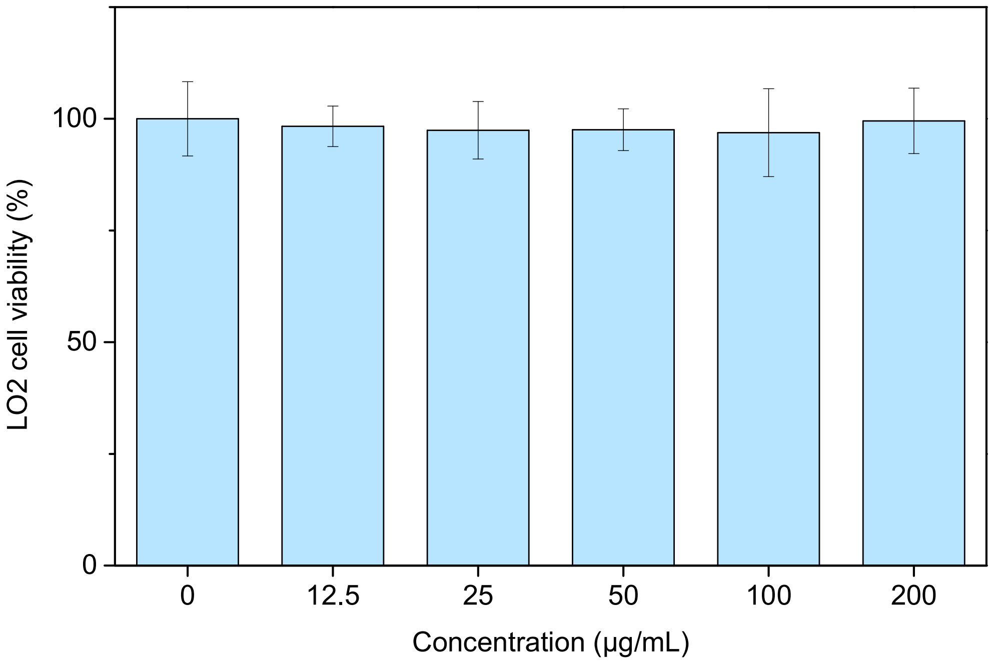

Figure S10. The cell viability of LO2 cells incubated with various concentrations of SSN for 24 h (n = 5).

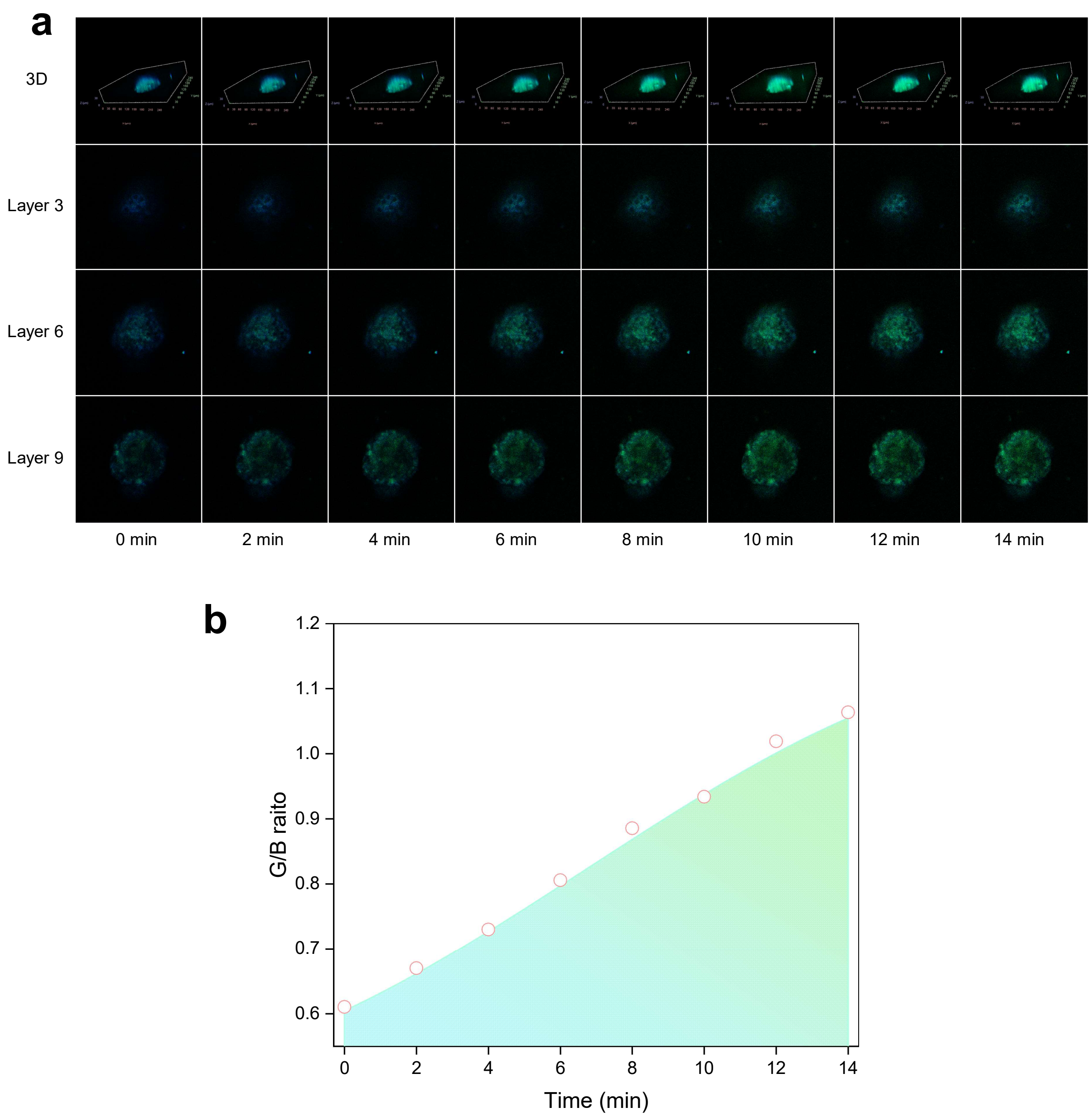

Figure S11. Confocal fluorescence images of multicellular spheroids treated with SSN+US for different time durations (**a**), and the corresponding ratio of green intensity to blue intensity (**b**).

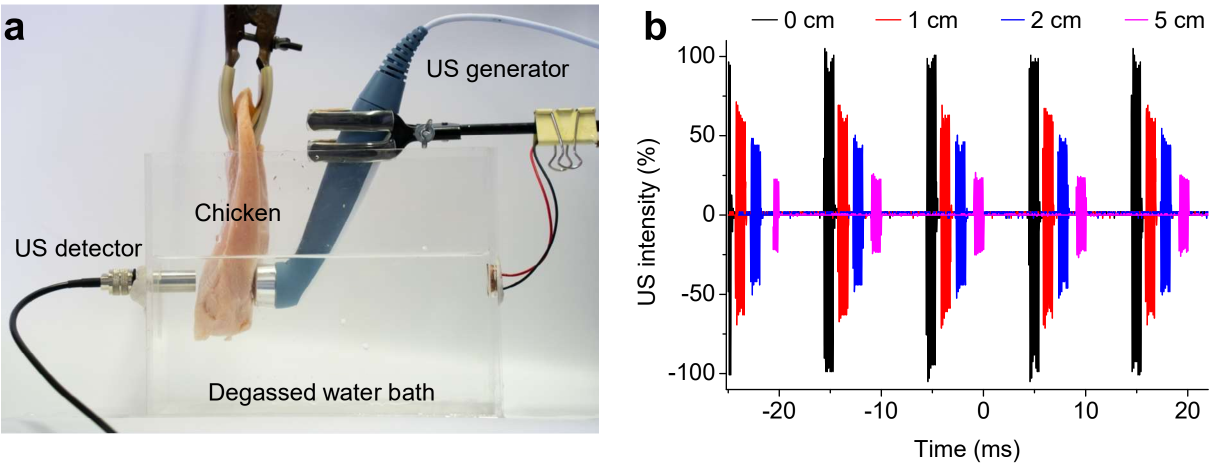

Figure S12. Ultrasonic penetration test of chicken in the degassed water (**a**), and obtained data in the absence (0 cm) and presence of chicken at different chicken thicknesses (1, 2, and 5 cm) (**b**).

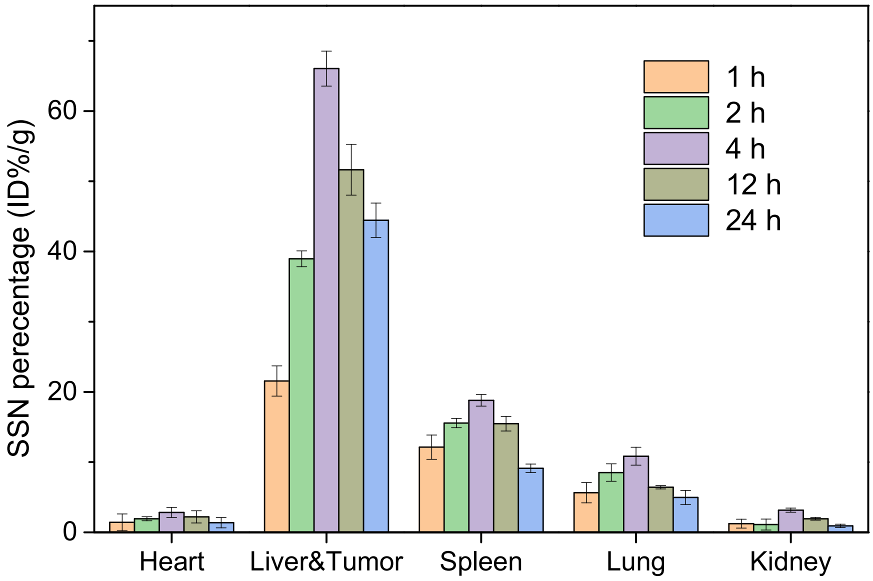

Figure S13. The biodistribution of SSN in the Hepa1-6 tumor-bearing mice after intravenous injection by the ICP measurement (n = 3).

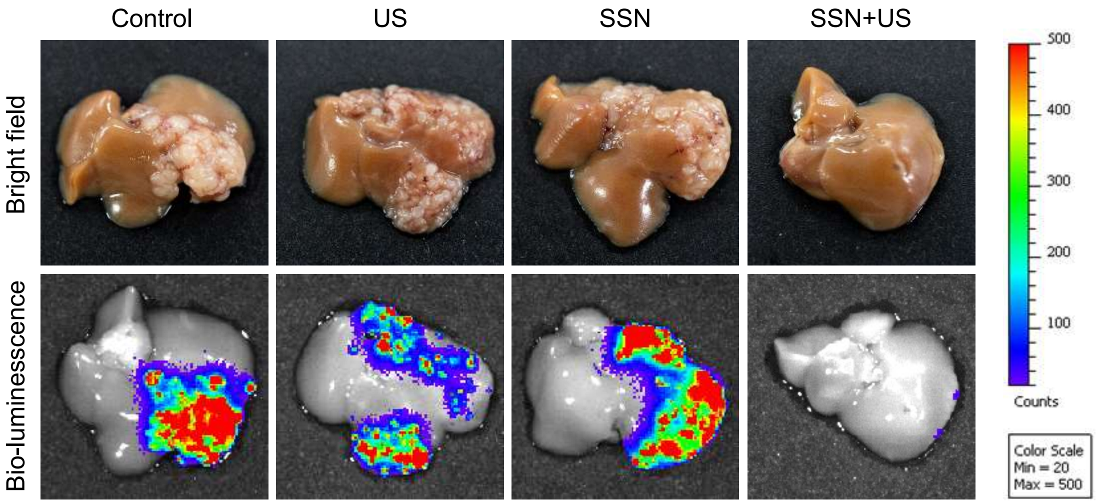

Figure S14. Representative digital photographs of the dissected tumors after 28 days of treatment.

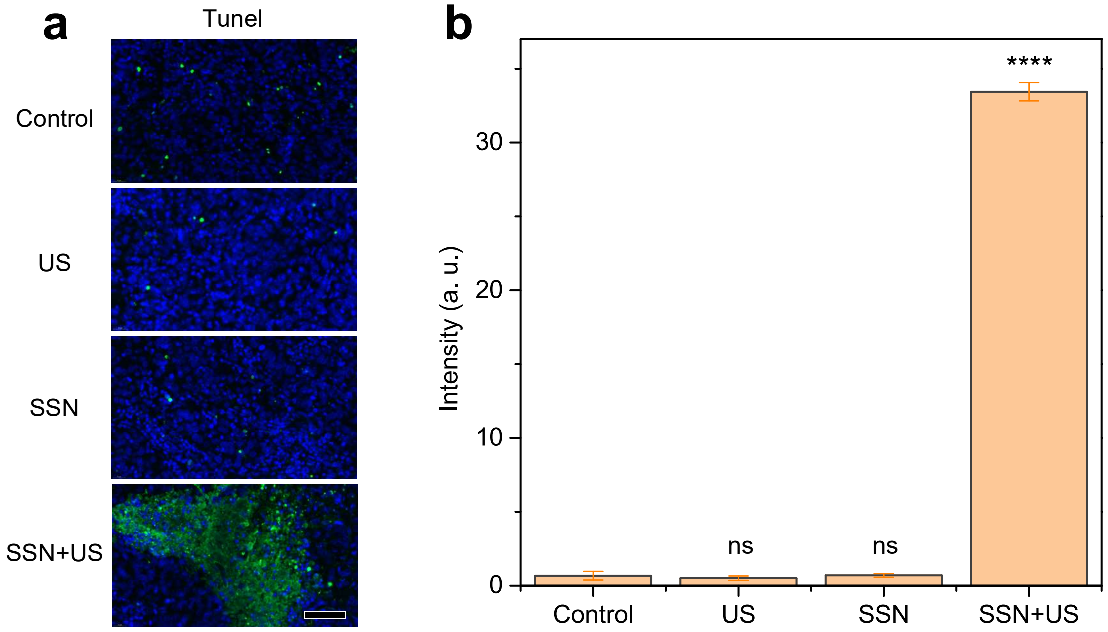

Figure S15. Immunohistochemical analysis of $TUNEL^+$ apoptotic cells after various treatments. *P* values were calculated by the one-way analysis of variance ANOVA method (*****p*<0.0001; ns, no significant difference). Scale bar, 50 μm.

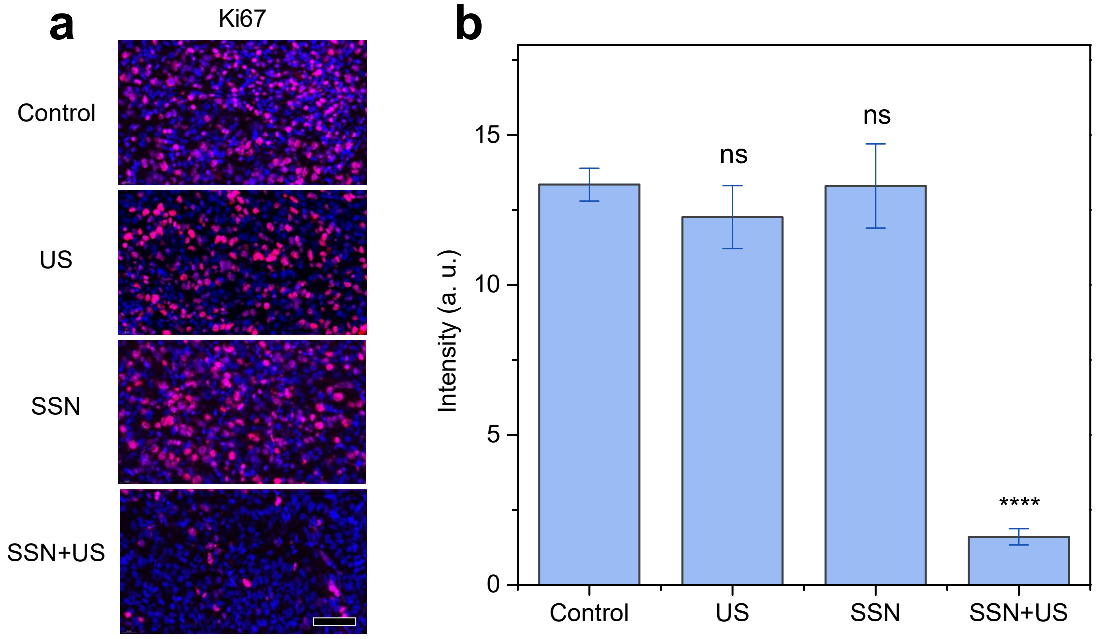

Figure S16. Immunohistochemical analysis of Ki67$^+$ proliferating cells after various treatments. P values were calculated by the one-way analysis of variance ANOVA method (****p<0.0001; ns, no significant difference). Scale bar, 50 μm.

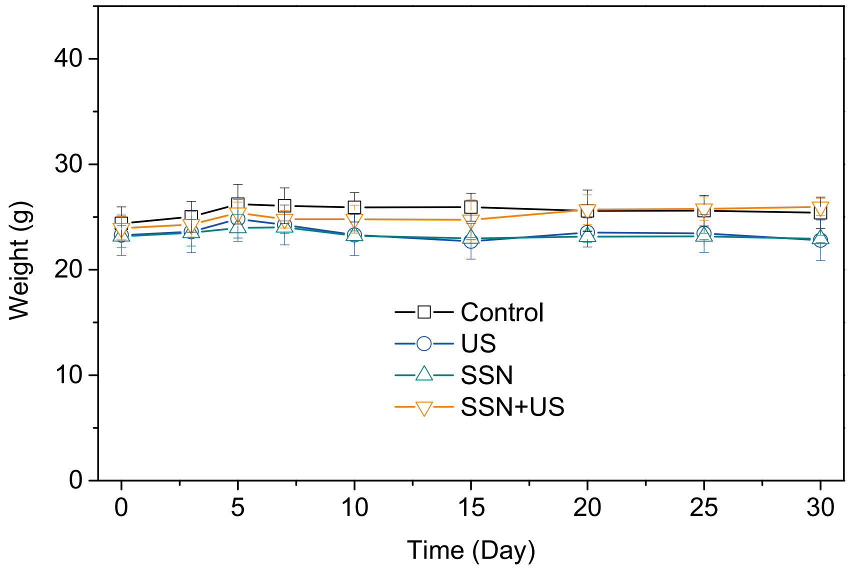

Figure S17. Body weight change of Hepa1-6-Luc tumor-bearing mice during various treatments (n = 7).

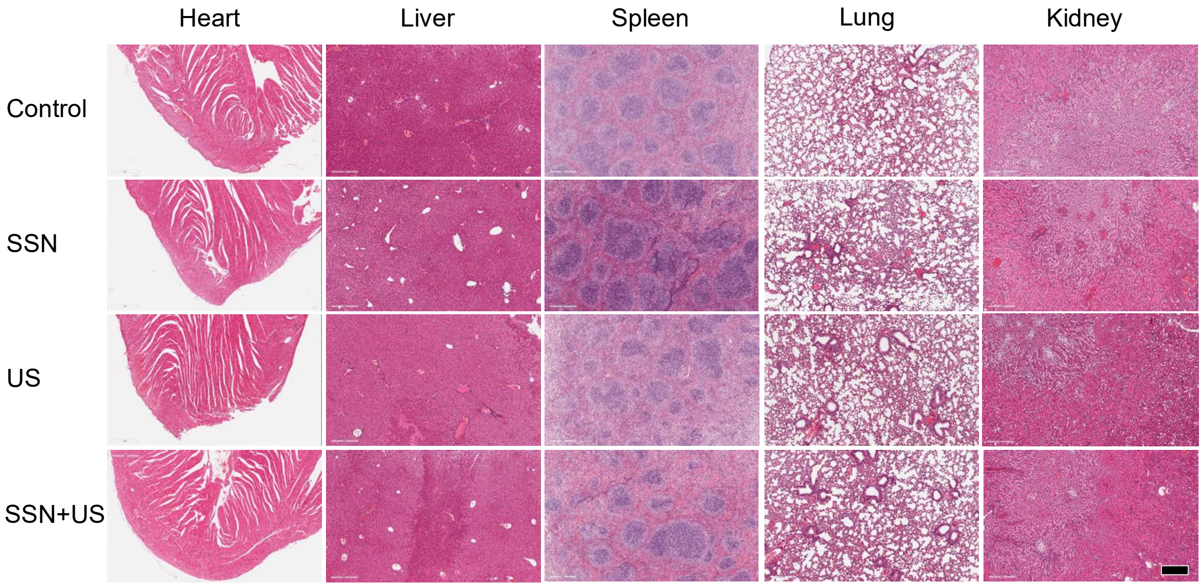

Figure S18. Histological examination of main organs (heart, liver, spleen, lung and kidney) from Hepa1-6 tumor-bearing mice after different treatments by the HE staining method. Scale bar, 300 μm.

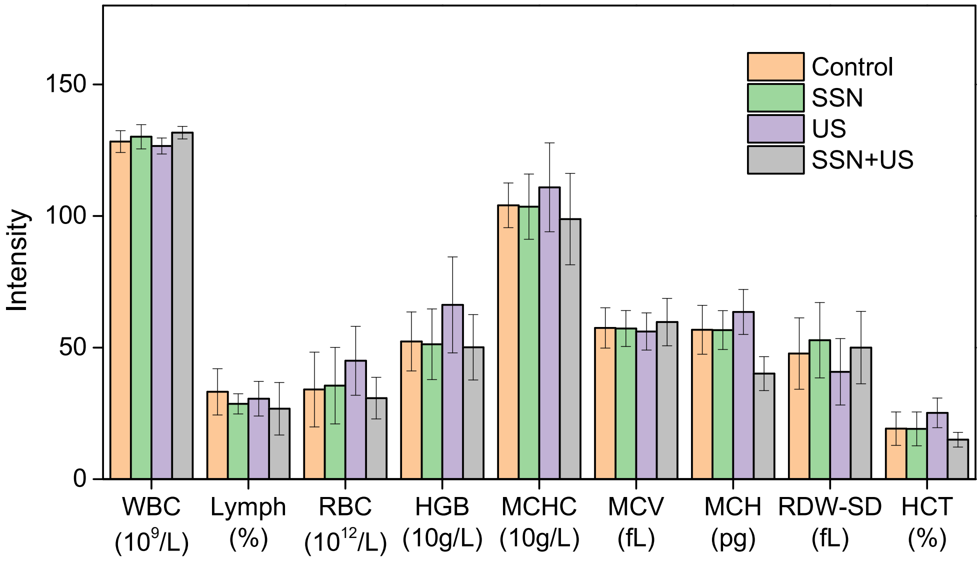

Figure S19. The assessment of standard haematological markers (n = 3) including white blood cells (WBC), red blood cells (RBC), hemoglobin (HGB), hematocrit (HCT), mean corpuscular volume (MCV), mean corpuscular hemoglobin (MCH), mean corpuscular hemoglobin concentration (MCHC), red blood cell volume distribution width (RDW-SD) and lymphocytes percentage (LYM).

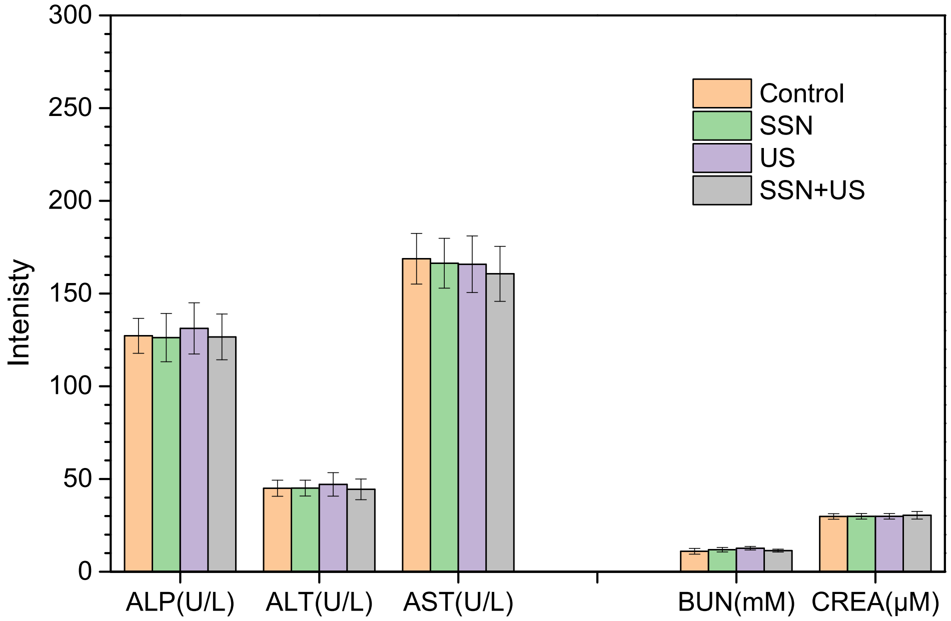

Figure S20. Blood biochemical analyses of liver/kidney functions of treated mice (n = 3).
